# Influence of Electrolytes on the Air–Water Interfacial Properties of Perfluoroalkyl Acids (PFAAs)

**DOI:** 10.1021/acs.langmuir.5c04005

**Published:** 2026-02-23

**Authors:** Muchu Zhou, Hayden B. McCray, Bor-Jier Ben Shiau, Brian P. Grady, Reza Foudazi

**Affiliations:** † School of Sustainable Chemical, Biological and Materials Engineering, The University of Oklahoma, Norman, Oklahoma 73019, United States; ‡ Chemical Sciences Division, Oak Ridge National Laboratory, Oak Ridge, Tennessee 37831, United States; § Mewbourne School of Petroleum and Geological Engineering, 6187 The University of Oklahoma, Norman, Oklahoma 73019, United States

## Abstract

Perfluoroalkyl acids (PFAAs), a subclass of per- and polyfluoroalkyl substances (PFAS), are widely used but pose significant environmental concerns due to their toxicity and bioaccumulation. Foam fractionation, utilizing the amphiphilic nature of PFAS, offers a promising remediation method by exploiting their migration to air–water interfaces. The effectiveness of this technique is highly dependent on the air–water interfacial properties and the adsorption capacity of PFAS at the interface. This study investigates the impact of electrolytes prevalent in PFAS-contaminated water on the air–water interfacial properties of PFAAs, focusing on surface tension and diffusion behavior. Our results show that electrolytes reduced surface tension for both long- and short-chain PFAAs, with divalent ions (Ca^2+^) exhibiting stronger effects than monovalent ions (Na^+^). Surface tension modeling using Gibbs and Extended Langmuir isotherms revealed enhanced adsorption and increased surface excess concentrations in the presence of electrolytes, while dynamic surface tension analysis highlighted the influence of electrolytes on molecular diffusion and adsorption kinetics at short time scales.

## Graphical abstract

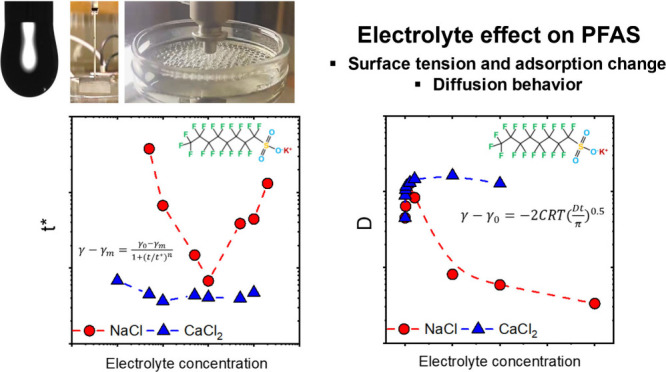

## 1 Introduction

Per- and polyfluoroalkyl substances (PFAS) are a group of synthetic chemicals extensively used in various industries due to their outstanding resistance to heat, chemical stability, and ability to repel water and oil. These uses include, but are not limited to, nonstick surfaces, surfactants, cosmetics, aqueous film forming foam (AFFFs), food packaging materials, and electronics.^1–4^ However, the bioaccumulation and toxicity of PFAS have raised significant health concerns.^5–8^ Exposure to these chemicals is linked to a variety of health risks, including liver and kidney diseases, obesity, reproductive issues, elevated cholesterol levels, disturbances in lipid and insulin metabolism, and an increased risk of cancer.^9–12^

Although some long-chain PFAS have limited solubility in the water, they are still detected in water sources all over the world.^10,13,14^ The United States Environmental Protection Agency (U.S. EPA) issued Maximum Contaminant Levels (MCLs) and Maximum Contaminant Level Goals (MCLGs) for perfluorooctanesulfonic acid (PFOS) and perfluorooctanoic acid (PFOA) in drinking water at 4 and 0 ng/L, respectively, in 2024.^15^ The MCLs were also set for several short-chain PFAS, for instance, 10 ng/L for perfluorohexanesulfonic acid (PFHxS), perfluorononanoic acid (PFNA), and hexafluoropropylene oxide (HFPO) dimer acid and its ammonium salt (GenX).^15^ Despite PFAS contamination in drinking water, industrial facility discharge (e.g., semiconductor manufacturing) can be several orders of magnitude higher in PFAS concentration than that of drinking water sources.^16^

Perfluoroalkyl acids (PFAAs) represent a significant subset of PFAS and are often highlighted for their prevalence and toxicity. These compounds are a focal point in research and regulatory efforts addressing PFAS due to their persistence and widespread use across various applications. A key factor driving the attention toward PFAAs is their role as the ultimate degradation products of PFAS; most PFAS, regardless of their initial form, eventually degrade into short-chain or long-chain PFAAs.^17,18^ This transformation underscores the environmental and health concerns associated with PFAAs, reinforcing their importance in both academic studies and governmental regulations related to PFAS management. PFOS and PFOA are the most representative species of PFAAs.

PFAAs typically comprise a hydrophilic headgroup and a hydrophobic, oleophobic tail that is partially or fully fluorinated, granting them unique surface-active behavior. Because of the amphiphilicity of PFAAs, which allows them to adsorb onto air–water interfaces, foam fractionation or aeration is a promising remediation method that can be used to remove PFAS from contaminated water.^19–33^ For example, Meng et al.^20^ utilized a lab-scale foam column to remove PFAAs including PFOS, PFOA, PFHxS, and perfluorobutanesulfonic acid (PFBS) from water with an improved removal efficiency when increasing the air injection rate and foaming time. Enhanced PFAS removal efficiency has been frequently reported when introducing electrolytes, i.e., salts. The removal of PFOS in the presence of 0–5 mM NaCl was tested by Meng et al.,^20^ demonstrating that the removal efficiency increases with increasing NaCl concentration. However, the advantage of adding NaCl on improving PFOS removal efficiency is mitigated if aerating for more than 2 h. Morrison et al.^27^ investigated the effect of temperature and salinity on PFAA removal efficiency, showing that adding NaCl to an AFFF solution and decreased temperature enhanced PFAA removal efficiency. In addition, the concentration of NaCl affects PFAA removal efficiency, for instance, the removal percentage of PFBS is improved from 25% to 60% during the aeration of 60 min when increasing the NaCl concentration from 0.113 to 0.599 M. The effect of metallic activators, such as Fe^3+^ and Ca^2+^, on PFAA removal efficiency was investigated by Lee et al.,^19^ indicating that the valence of ions influences the removal efficiency, for example, the highest PFOS removal efficiency of 99.5% was obtained when introducing Fe^3+^. Moreover, Hu et al.^30^ added whey soy protein to an aeration system to remove 5–20 ppm PFOS or PFOA with high recovery rates of 94–98% at a pH of 6 when introducing >200 ppm of whey soy protein due to the reduced surface tension and binding between whey soy protein and PFOS/PFOA. Hu et al.^30^ pointed out that liquid drainage occurring in the foam is intensified, therefore, higher enrichment of PFOS and PFOA since the amount of recovered water is reduced.

Although high removal efficiency up to 99% was reported by employing foam fractionation, one challenge is to efficiently remove short-chain PFAS, i.e., PFBS and perfluorobutanoic acid (PFBA).^20,27,31^ Recently, Lee and Venkatesan^29^ studied the removal of PFBS and PFBA using foam fractionation in the presence of cosurfactant cetyltrimethylammonium chloride (CTAC) and electrolytes. It was found that adding 10 mM NaCl or CaCl_2_ was not helpful to remove either PFBS or PFBA. According to our recent study,^34^ a maximum salt concentration exists beyond which the foaming capacity decreases. However, the addition of salts (NaCl, CaCl_2_, and Na_2_SO_4_) was found to enhance the removal of PFBS in the presence of CTAC.^29^ Nonetheless, the effect of adding multivalent salts on PFAS adsorption onto air–water interfaces has rarely been fundamentally studied. Also, despite many studies investigating the PFAS removal efficiency in the presence of salt ions, systematic studies on the correlation between the PFAS foaming behavior and their interfacial properties in the presence of electrolytes are still limited.

Despite the investigation on PFAA removal by using foam fractionation, air–water interfacial properties are crucial to study since the foaming behavior of PFAAs is highly affected by their interfacial properties through influencing the stability of the foam films.^34–37^ For example, foam height, foam stability, and length of lamellae are influenced by the maximum rate of surface tension reduction, ${\left(\frac{d\gamma_{t}}{dt}\right)}_{\max}$ .^38,39^ In addition to surface tension, interfacial rheology is another important factor influencing the foaming properties of PFAAs since it was reported that films tend to be more stable if they have a higher interfacial elasticity.^35^ According to Wang et al.’s work,^40^ the foaming capacity of surfactant-stabilized aqueous foams is dependent on their dilatational interfacial modulus. High interfacial elasticity at low frequencies reduces Ostwald ripening of gas bubbles resulting in high foam stability.^41^ High interfacial elasticity at high frequencies reduces bubble coalescence and thereby increases foam capacity.^41^ Another study found that foam stability was reported to be governed by the viscoelastic modulus rather than molecular diffusion.^42^ Our previous work suggested that at the same molar concentration, short chain PFAAs did not show a dilatational modulus within our ability to measure in a pendant drop due to low adsorption density.^37^ Our previous study on PFAA foaming properties demonstrated the significance of interfacial properties represented by dimensionless numbers, such as Capillary, Weber, Boussinesq and interface numbers, on their foaming behavior.^34^ Beyond interfacial parameters, diffusivity is also a parameter that warrants further investigation, as it can be used to determine mass transfer coefficients within a foam fractionation system.

In this present work, the air–water interfacial properties of PFAAs are investigated in the presence of electrolytes (NaCl and CaCl_2_) with various concentrations to better understand how electrolytes interact with PFAAs and influence their behavior at air–water interfaces. Three typical PFAAs, PFOS, PFOA and PFBS, are used in this study. Surface tensions of the PFAA solutions with and without addition of salts are measured by using different tensiometries, and the Gibbs and Extended Langmuir isotherms are employed to find the maximum concentration of PFAAs at the air–water interface. Quantitative insights into short-term dynamic surface tension and diffusion behaviors across a wide range of electrolyte concentrations are provided. Reported surface tension values for PFAAs can vary significantly between studies, and it is not clear if this variation is due to experimental or systematic error. By measuring surface tension with multiple types of tensiometries, we can identify potential systematic errors that are inherent to a given methodology. In addition to the surface tensions, the diffusion coefficients of PFAAs in water are estimated to study the effect of introducing salts. The authors are not aware of other studies that have comprehensively examined interfacial phenomena as well as diffusivity of the PFAAs considered herein.

## 2 Experimental Section

### 2.1 Materials

KPFOS (heptadecafluorooctanesulfonic acid potassium salt, purity ≥98.0%, CAS: 2795-39-3), PFOA (perfluorooctanoic acid, purity 95%, CAS: 335-67-1), and KPFBS (potassium nonafluoro-1-butanesulfonate, purity 98%, CAS: 29420-49-3) were purchased from Sigma-Aldrich. Figure 1 depicts the structures of KPFOS, PFOA, and KPFBS. Sodium chloride (NaCl) was purchased from Fisher BioReagents (purity ≥99.0%, CAS: 7647-14-5), and calcium chloride (CaCl_2_) was purchased from Thermo Scientific (purity 97%, CAS: 10043-52-4). All chemicals were used as received without further purification, although recent work has established that the effect of impurities can be prominent at low ionic strengths for mixtures of PFAS.^43^ Ultrapure deionized water purified by a Millipore Synergy Water Purification System was used to prepare all PFAA aqueous samples. PFAA stock solutions were prepared and stored in polypropylene bottles for future use. The specifics of the PFAA aqueous solutions prepared for this investigation are detailed in Table 1.

**Figure 1:**
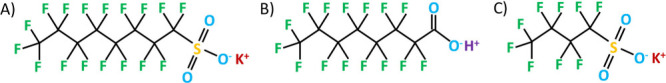
Chemical structure of (A) KPFOS, (B) PFOA, and (C) KPFBS.

**Table 1:** Sample Formulations

	PFAA concentration range (mM)	focus PFAA concentration (mM)	salt	salt concentration (mM)
KPFOS	0.0002–1.11	0.40	NaCl	0.5, 1, 5, 10, 50, 100, 200
			CaCl_2_	0.1, 0.5, 1, 5, 10, 50, 100

PFOA	0.0002–10	0.40	NaCl	0.5, 1, 5, 10, 50, 100, 200
			CaCl_2_	0.1, 0.5, 1, 5, 10, 50, 100
				
KPFBS	0.0003–88.71	0.40	CaCl_2_	0.1, 100

### 2.2 Surface Tension Measurement

#### 2.2.1 Wilhelmy Plate Tensiometry

Using a Wilhelmy plate tensiometer (Dataphysics, DCAT 25), the surface tension of PFAA aqueous solutions over a period of 1 h was assessed at 25 ± 0.5 °C. Pre-experimental procedures included tensiometer calibration and comprehensive cleansing of the Wilhelmy plate with a Bunsen burner, emphasizing the importance of maintaining a pristine testing environment. At least 2 replicates were measured. An extrapolation was used to obtain the equilibrium surface tension, the details as well as the uncertainty calculations can be found in our previous work.^37^

#### 2.2.2 Pendant Drop Tensiometry

To further investigate the interfacial properties of PFAAs in an air–water environment, specifically focusing on PFOA, an optical tensiometer (Attension KSV Instruments, Biolin Scientific, Finland) was employed. This experiment involved observing either a pendant drop of the aqueous solution suspended in air or an air bubble generated within the PFOA solution. To generate the air bubble, a J-shaped needle (Dataphysics, 6000105, SNC 107/069 Dosing needle: Outer Ø 1.07 mm; Inner Ø 0.69 mm; Length 57 mm; Width 10 mm; Upward 7 mm) was utilized, a procedure detailed in Figure S1. Each sample composition was subjected to a minimum of two replicate tests to ensure the accuracy and reliability of the results. The Young–Laplace equation was used for drop shape curve fitting to obtain the surface tension.^37,44,45^

#### 2.2.3 Bubble Pressure Tensiometry

To assess the rapid adsorption dynamics of PFAAs at air–water interfaces, a bubble pressure tensiometer (KRÜSS, BP100) equipped with a capillary tube with a diameter of 0.228 mm was used to measure the dynamic surface tension on a very short time scale. A minimum volume of 70 mL of PFAA solution was placed in a glass vessel (VWR 216-0067, 70 mm, Boro 3.3). A crucial step in the preparation involved cleaning the capillary tube with ultrapure deionized water before each measurement.

## 3 Results and Discussion

### 3.1 Surface Tension

Figure 2 shows and summarizes the equilibrium surface tension of KPFOS, PFOA, and KPFBS at various bulk concentrations with and without adding electrolytes in our work and from previous work.^21,46–53^ Equation 1 describes the dynamic surface tension, γ, and indicates that the equilibrium surface tension, γ_e_, is obtained by extrapolating a linear plot of γ versus *t* ^−0.5^ to *t* ^–0.5^ → 0.^54,55^

1 $$\gamma -\gamma_{e}=\frac{2RT\Gamma_{m}^{2}}{C}{\left(\frac{1}{\pi D_{\mathrm{eff}}t}\right)}^{0.5}$$

In this equation, *R* is the gas constant, *T* is the temperature in Kelvin, Γ_m_ is the maximum surface excess concentration, *C* is the surfactant bulk concentration, *D*_eff_ is the effective diffusion coefficient, and *t* is time. The *γ*_e_s of 0.4 mM PFAAs are summarized in Table 2. The details of uncertainty calculations can be found in SI and Table S1.^37^ The surface tensions of PFAAs are reduced when introducing NaCl, and the divalent salt CaCl_2_ reduces the surface tension further. Qazi et al.^56^ observed surface tension reduction of cationic surfactant hexadecyltrimethylammonium bromide (CTAB) upon an addition of NaCl due to interfacial charge screening. Notably, for both KPFOS and PFOA, 10 mM CaCl_2_ leads to a greater reduction in surface tension than 100 mM NaCl. Since the 10 mM CaCl_2_ systems have a lower overall ionic strength than the 100 mM NaCl systems, this behavior cannot be explained by the salting-out effect alone. Instead, the enhanced surface tension reduction suggests more effective electrostatic screening, likely due to the stronger binding affinity of Ca^2+^ for the anionic headgroups of KPFOS and PFOA compared to Na^+^.

**Figure 2:**
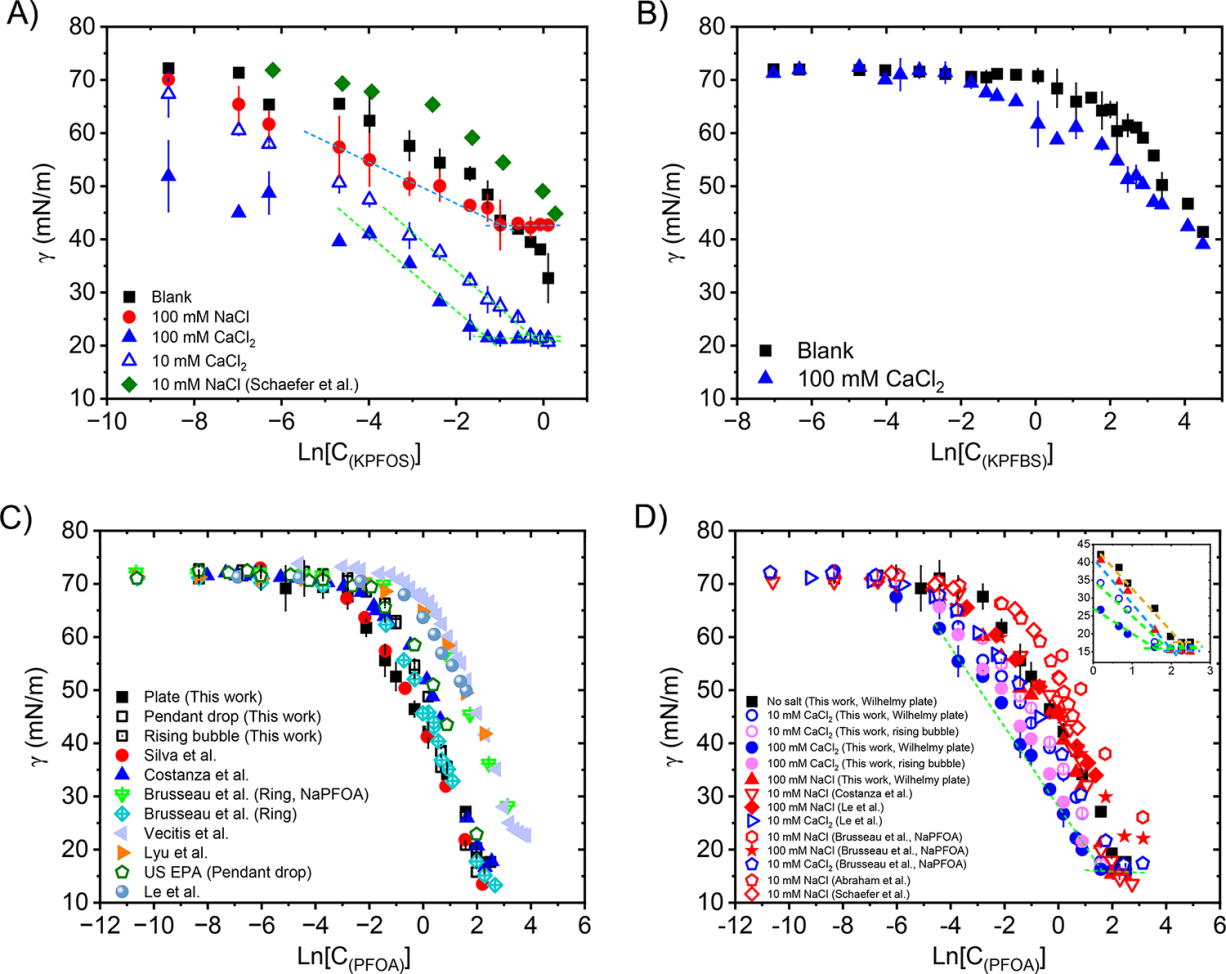
(A) KPFOS surface tension versus concentration (mM) in the presence of 100 mM NaCl, 10 mM, and 100 mM CaCl_2_. The black solid square points are the samples without the addition of electrolytes, and the green solid points are from Schaefer et al.,^46^ showing the surface tension of KPFOS in the presence of 10 mM NaCl. (B) KPFBS surface tension versus concentration (mM) with and without adding 100 mM CaCl_2_. (C) PFOA or NaPFOA surface tension versus concentration (mM) in this study and from the literature.^21,47–52^ (D) PFOA (NaPFOA) surface tension versus concentration (mM) in the presence of 10 mM and 100 mM NaCl, and 10 mM and 100 mM CaCl_2_ conducted in this study and retrieved from the literature.^46,48,49,51,53^ The inset shows the surface tension region corresponding to the CMC of PFOA with and without adding electrolytes.

**Table 2:** Properties of PFAAs in the Present Study (Γ_m,G_ is Obtained from the Gibbs Isotherm, and Γ_*m*,EL_ is Derived from the Extended Langmuir isotherm)

PFAS	salt	molecular weight (g/mol)	solubility in water (mM)	CMC (mM)	γ_e_ (mN/m) for 0.4 mM aqueous solution at 25 °C	Γ_m,G_ (molecule/nm^2^)	Γ_m,EL_ (molecule/nm^2^)
KPFOS		538.22	∼1.1–1.2, 0.93,^67^ 1.06 ^60^	7,^59^ 8,^60^ 8.5 ^61^	43.540 ± 2.648 ^37^	1.18 ± 0.11 ^37^	1.98 ± 0.12 ^37^
	100 mM NaCl			∼0.4	42.714 ± 4.608	1.29 ± 0.03	3.01 ± 9.98
	10 mM CaCl_2_			∼0.9–1.0	27.317 ± 1.790	1.59 ± 0.04	3.15 ± 2.91
	100 mM CaCl_2_			∼0.2	21.175 ± 1.234	1.83 ± 0.10	9.23 ± 1.68

PFOA (P.d.^a^)		414.07			62.542 ± 0.095	1.99 ± 0.10	4.59 ± 0.36

PFOA (R.b.^b^)					63.071 ± 0.329	1.68 ± 0.09	3.35 ± 0.24

PFOA			∼9.7	∼9.7	52.562 ± 2.603	1.16 ± 0.05	1.88 ± 0.09
	100 mM NaCl			∼7.3	49.115 ± 0.405	3.25 ± 0.14	7.23 ± 0.30
	10 mM CaCl_2_			∼7.3	43.488 ± 0.330	2.23 ± 0.11	3.88 ± 0.20
	100 mM CaCl_2_			∼4.8	37.848 ± 0.144	2.15 ± 0.04	3.81 ± 0.01

KPFBS		338.19	136.61 ^67^	22 ^59^	71.160 ± 0.737 ^37^	1.16 ± 0.22 ^37^	2.34 ± 0.13 ^37^
	100 mM CaCl_2_				66.912 ± 1.293	1.59 ± 0.06	2.02 ± 0.09

a Pendant drop tensiometry and pendant drop method.

b Rising bubble method.

In addition to surface tension reduction, electrolytes also influence the critical micelle concentration (CMC) of PFAS by reducing headgroup repulsion.^57,58^ The CMC of KPFOS is around 7–8.5 mM,^59–61^ which is far above its solubility limit (∼1.1–1.2 mM), and thus, the CMC of KPFOS is not observed in Figure 2A. However, in the presence of electrolytes, the CMC of KPFOS shifts to lower concentrations. In the presence of 100 mM NaCl, the CMC is ∼0.4 mM, while the CMC is ∼0.2 mM in the presence of 100 mM CaCl_2_. The concentration of electrolytes also affects the CMC of KPFOS, for example, a higher CMC of around 1 mM is obtained if only adding 10 mM CaCl_2._ For KPFBS, the CMC is still not observed upon adding 100 mM CaCl_2_. Furthermore, the solubility limit of PFOA is approximately 9.7 mM (4000 ppm), which is near its CMC. Upon the addition of electrolytes, the PFOA CMC also shifts to lower concentrations (Figure 2D). A CMC of 7.3 mM is obtained when introducing 100 mM NaCl into the PFOA aqueous solutions, while the CMC is 4.8 mM in the presence of 100 mM CaCl_2_. If the CaCl_2_ concentration is decreased to 10 mM, the CMC is around 7.3 mM. Steffens et al.^58^ evaluated the composition of 3 M AFFF, and reported that high salinity reduces the CMC of the AFFF and increases the amount of PFAS at the interface.

In addition to the CMC, the micellization behavior of PFAS in water is affected by the addition of electrolytes through influencing the Gibbs free energy of micellization (Δ*G*_mic_). The related equations can be found in SI. Δ*G*_mic_ becomes more negative by adding electrolytes; for instance, Kancharla et al.^57^ reported the CMC shifts for NaPFO and NaPFHx (sodium salts of PFOA and perfluorohexanoic acid, respectively) in the absence and presence of NaCl. They demonstrated that the lower CMCs of NaPFO is due to their longer fluorinated chains, giving rise to a more negative Δ*G*_tr_ (transfer free energy), lower Δ*G*_int_ (interfacial free energy), and lower Δ*G*_pack_ (packing free energy).^57^ According to their work,^57^ the micellization of long-chain PFAS is influenced more by adding NaCl compared to that of short-chain PFAS due to the greater decrease in the entropy lost by binding the counterions onto the charged micelle surface after adding NaCl. Moreover, a greater increase in Δ*G*_elec_ (electrostatic free energy) with the addition of NaCl is obtained. Additionally, small angle neutron scattering (SANS) shows an increase in micelle size in the presence of 0.25 M NaCl.^57^

In addition to the Wilhelmy plate method, pendant drop and rising bubble methods were also used for measuring the surface tension of PFOA. Figure S2 presents the pendant drop and rising bubble of 0.4 and 2.4 mM PFOA aqueous solutions with and without salts, respectively. Based on our results, the middle surface tension regime has higher apparent values with the Wilhelmy plate method compared to pendant drop/rising bubble. Nevertheless, in the lower and higher surface tension regimes, the effect of method becomes less significant.

Equation 2 is the Gibbs isotherm,^37,55,62^

2 $$\Gamma =-\frac{1}{nRT}{\left(\frac{d\gamma }{d\ln C}\right)}_{T}$$

where *n* = 1 for surfactants (nonionic or ionic in the presence of electrolytes) and *n* = 2 for ionic surfactants without the addition of swamping electrolytes, and Γ is the surface excess concentration.^63^ The Gibbs isotherm can be used to estimate the surface excess concentration of PFAS as a function of bulk PFAS concentration *C*. One approach to calculate the derivative is to first fit the surface tension below the CMC with the polynomial,^62^ and then apply the Gibbs isotherm. The fitting method was described in our previous work.^37^ Figure S3 shows the fitting example of KPFOS in the presence of 100 mM CaCl_2_ with all the fitted parameters listed in Tables S2–S3. Figure 3 presents the local surface excess concentrations of PFAS with and without the addition of electrolytes. Figure 3A shows that NaCl increases the surface excess of KPFOS until the bulk KPFOS concentration is above approximately 0.3 mM. Furthermore, surface excess of KPFOS is generally higher with CaCl_2_ than NaCl with the exception to this observation for bulk KPFOS concentrations below 0.02 mM. CaCl_2_ increases the surface excess of KPFBS until the bulk KPFBS concentration is approximately 90 mM (Figure 3B). NaCl and CaCl_2_ both increase the surface excess of PFOA as shown in Figure 3C. Surface excess of PFOA is nearly identical for 100 mM NaCl and 10 mM CaCl_2_, whereas the surface excess of PFOA with 100 mM CaCl_2_ is higher until the bulk PFOA concentration is approximately 2 mM where a maximum is observed. The similar trends observed for KPFOS and PFOA indicate increased surface excess in CaCl_2_ systems relative to NaCl, which attributes to the higher binding affinity and stronger electrostatic interactions between Ca^2+^ ions and the anionic sulfonate and carboxylate headgroups.

**Figure 3:**
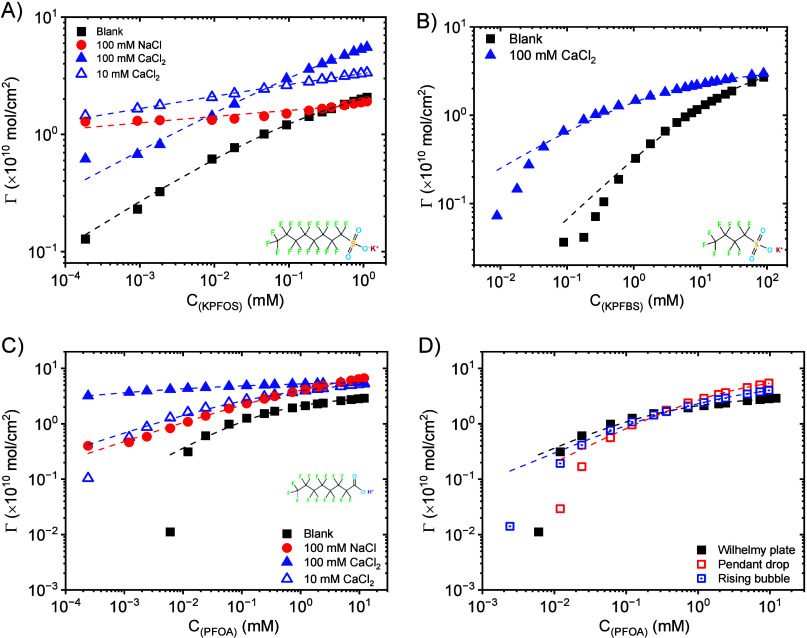
(A) Surface excess concentration of KPFOS with and without adding electrolytes (NaCl and CaCl_2_) as a function of KPFOS bulk concentration. (B) Surface excess concentration of KPFBS with and without adding CaCl_2_ as a function of KPFBS bulk concentration. (C) Surface excess concentration of PFOA with and without adding electrolytes (NaCl and CaCl_2_) as a function of PFOA bulk concentration. (D) Comparison of PFOA surface excess concentrations obtained by using Wilhelmy plate tensiometry, pendant drop tensiometry, and rising bubble tensiometry. The dashed lines are the Extended Langmuir isotherm fit.

The Langmuir isotherm is shown as equation 3:^37,55,64^

3 $$\Gamma =\Gamma_{m}\frac{K_{L}C}{1+K_{L}C}$$

where *K*_L_ is the equilibrium Langmuir adsorption constant and Γ_m_ is the maximum surface excess concentration. The Langmuir isotherm is an empirical model based on kinetic principles, that is the adsorption and desorption rates are equal with zero accumulation at equilibrium conditions.^64^ The adsorption curve of Langmuir isotherm is a L-shape, however, an S-shaped adsorption isotherm was proposed, known as the Extended Langmuir isotherm (Sips isotherm):^37,64–66^

4 $$\Gamma =\Gamma_{m}\frac{K_{L}C^{n}}{1+K_{L}C^{n}}$$

where *K*_L_ is the equilibrium constant of the surface aggregation process, and *n* is the average aggregation number of the surface aggregate. Since the Extended Langmuir isotherm generates more accurate fits based on the *R* ^2^ according to our previous work,^37^ only eq 4 is used in this study.

The dashed lines presented in Figure 3 are from the Extended Langmuir isotherm fit to the data with Table S4 summarizing the fitted parameters. Γ_m_s of PFAS are summarized in Table 2 where Γ_m,G_ and Γ_m,EL_ are derived from the Gibbs and Extended Langmuir isotherms, respectively. A higher value of Γ_m_ is obtained by applying the Extended Langmuir model. The Gibbs and extend Langmuir fittings generally demonstrate that the Γ_m_s of PFAS increase upon the addition of electrolyte. For example, the Γ_m_ of KPFOS is enhanced from 1.18 molecules/nm^2^ to 1.83 molecules/nm^2^ after adding 100 mM CaCl_2_ according to the Gibbs model. The one exception to the increase is KPFBS from the Extended Langmuir model. In addition, Γ_m_ is larger for PFOA when the salt is NaCl vs CaCl_2_ while the opposite is true for KPFOS. Furthermore, the PFOA Γ_m_s obtained by using three tensiometries are different at low surfactant concentrations due to differences in the measured surface tension. This result indicates that the selection of tensiometers has an impact on the measured value of the maximum surface excess concentration.

Figure 4 displays the concentration effect of electrolytes on the surface tension of PFAS. For KPFOS, the surface tension decreases with an increase in the concentration of the divalent salt (CaCl_2_), but this surface tension reduction trend was not significant for KPFOS with the monovalent salt (NaCl) within the experimental errors. This trend is less profound compared to the addition of CaCl_2_. For PFOA, the surface tension depends on both monovalent salt (NaCl) and divalent salt (CaCl_2_) concentrations, but only higher concentrations of monovalent salt (NaCl) affect the surface tension of PFOA. In the presence of NaCl, PFOA exhibits a clear decrease in surface tension with an increasing salt concentration, whereas KPFOS shows no such trend. Only CaCl_2_ was tested for KPFBS, and the surface tension of KPFBS is smaller at 100 mM salt concentration vs no salt as shown in Figure 2B. However, there is no clear change in surface tension with salt concentration shown in Figure 4; however, the error bars in Figure 4 are quite large.

**Figure 4:**
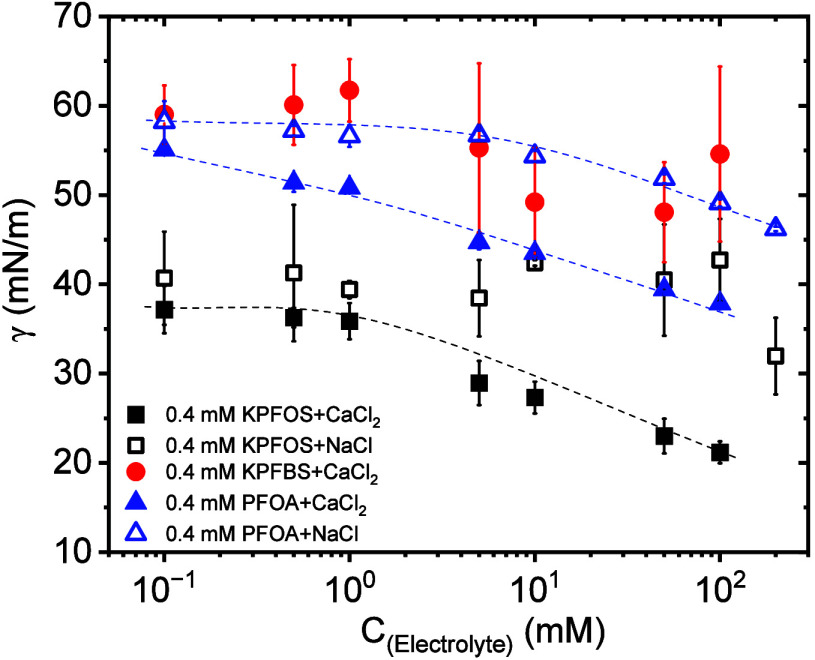
Effect of electrolyte concentration on the surface tension of PFAAs (the dashed lines are provided as a guide showing the trend).

### 3.2 Short-Time Adsorption of PFAAs

Figure 5 presents the dynamic surface tension (DST) of KPFOS at different bulk concentrations, both with and without electrolytes, over a short time scale. DSTs of KPFOS decrease with an increasing bulk concentration. In the presence of 100 mM NaCl, KPFOS DSTs are not significantly reduced; however, DSTs are dramatically decreased in the first 10 s when 10 mM and 100 mM CaCl_2_ are present. Typical DST curves usually display four stages: (1) induction where the surface tension does not change, (2) fast fall where the surface tension reduces quickly, (3) meso-equilibrium where the surface tension changes slowly toward an asymptotic equilibrium surface tension, and (4) equilibrium where the surface tension does not change.^68^ The equation below was proposed by Hua et al.^68^ for the first three stages:

5 $$\gamma -\gamma_{m}=\frac{\gamma_{0}-\gamma_{m}}{1+{\left(t/t^{*}\right)}^{n}}$$

where *γ*_m_ is the equilibrium surface tension in the stage of meso-equilibrium, γ_0_ is the surface tension in the absence of surfactants. t* is the characteristic decay time of the fast-fall stage, representing the time at which surface tension drops by half its overall change, and it has units of time; *n* is a dimensionless fitting parameter; Γ_e_, obtained from eq 1, was taken as *γ*_m_. The solid lines in Figure 5 are the fitting lines of eq 5 with parameters listed in Tables S5–S8.

**Figure 5:**
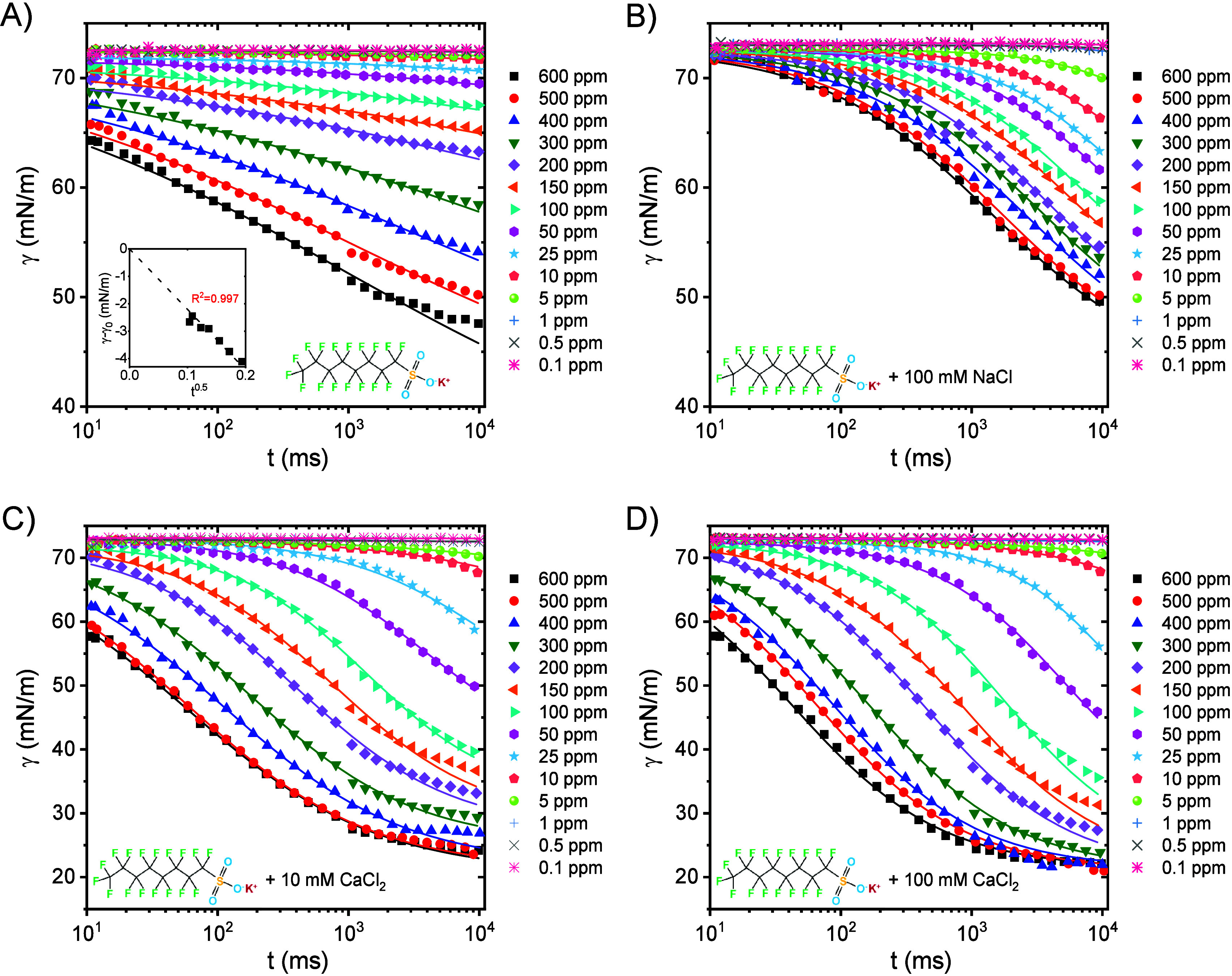
Dynamic surface tension measured with a bubble pressure tensiometer of (A) various concentrations of KPFOS, (B) various concentrations of KPFOS with an addition of 100 mM NaCl, (C) various concentrations of KPFOS with an addition of 10 mM CaCl_2_, and (D) various concentrations of KPFOS with an addition of 100 mM CaCl_2_.

For KPFOS, a fast fall stage is found for all concentrations (Figure 5A), but the induction stage becomes shorter with increasing KPFOS bulk concentrations. Additionally, the meso-equilibrium stage is not reached; i.e. the data collection time was not long enough to see a slowing of the change in surface tension with time. With 100 mM NaCl, the induction stage becomes longer versus no added electrolyte as seen from comparing Figure 5B with Figure 5A, even though γ_0_ – *γ*_m_ remains the same. When adding 100 mM CaCl_2_, the induction stage becomes shorter, γ_0_ – *γ*_m_ becomes larger, and at higher KPFOS concentrations (300–600 ppm), the meso-equilibrium stage is obtained (Figure 5D). Decreasing the concentration of CaCl_2_ to 10 mM, the induction stage is not influenced dramatically vs no added electrolyte, but the meso-equilibrium stage is reached and γ_0_ – *γ*_m_ slightly decreases (Figure 5C).

The Fickian diffusion coefficient can be approximated from eq 6 if short enough times are used to measure the surface tension:^54,55^

6 $$\gamma -\gamma_{0}=-2CRT{\left(\frac{Dt}{\pi }\right)}^{0.5}$$

where *D* is the Fickian diffusion coefficient. The inset of Figure 5A shows an example of calculating the *D* when plotting γ – γ_0_ vs *t* ^0.5^. Further discussion of *D* is provided in the next section.

Figure 6 shows the DSTs of PFOA at various bulk concentrations in the presence and absence of electrolytes. Solid lines are the model based on eq 5 with the fitted parameters summarized in Tables S9–S12. As with KPFOS, the induction stage becomes shorter as the bulk concentration of PFOA increases, as shown in Figure 6A. No further decrease of surface tension is observed above a PFOA concentration of ∼9.7 mM (4000 ppm), indicating the CMC. The CMC shifts to lower concentrations when adding electrolytes in agreement with the Wilhelmy plate tensiometer results. Upon an addition of electrolytes regardless of the valence, no significant change of induction stage time is observed and γ_0_ – *γ*_m_ increases. When adding 100 mM NaCl, less time is necessary to reach the meso-equilibrium stage compared with adding 100 mM CaCl_2_.

**Figure 6:**
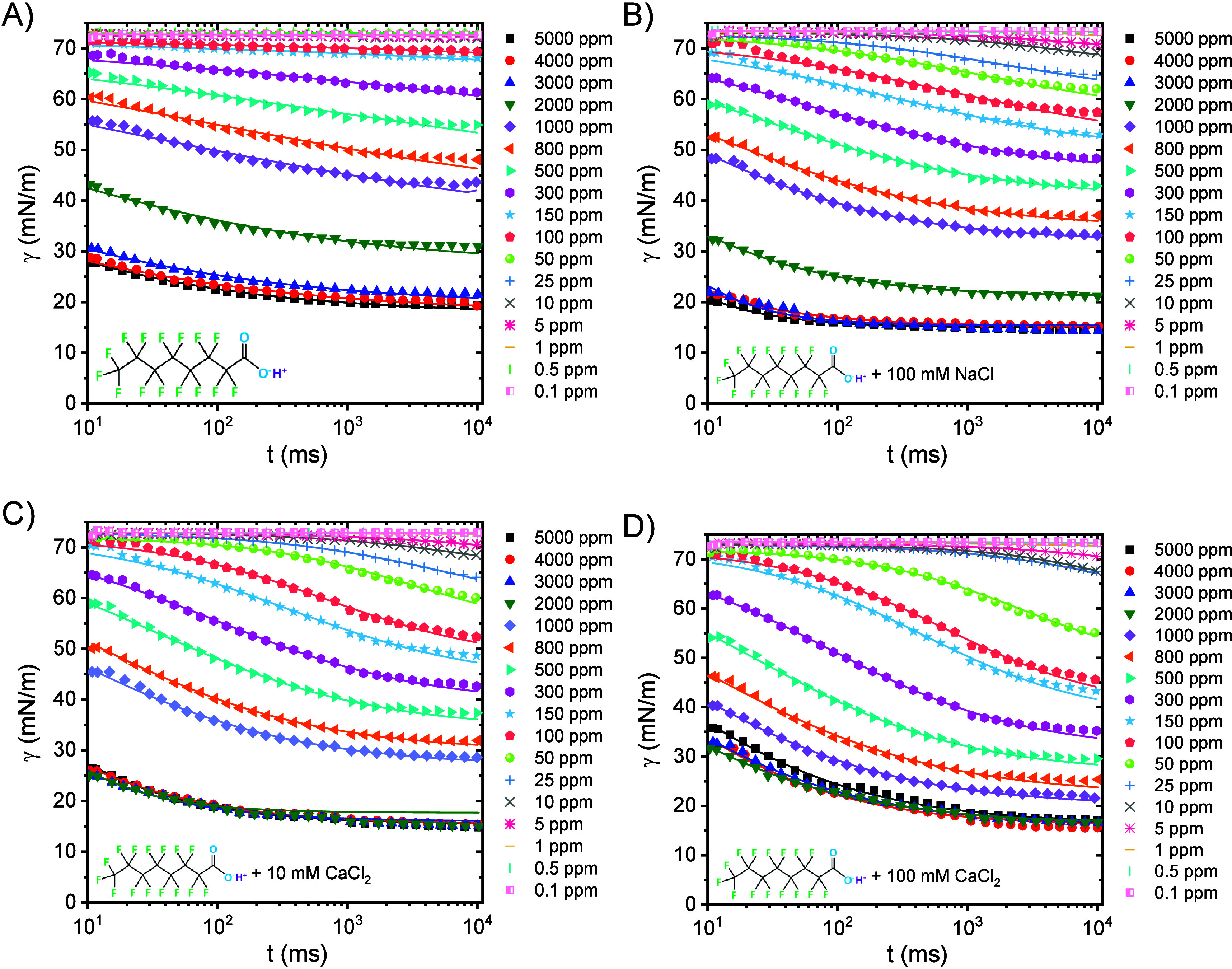
DSTs measured with a bubble pressure tensiometer of (A) various concentrations of PFOA, (B) various concentrations of PFOA with an addition of 100 mM NaCl, (C) various concentrations of PFOA with an addition of 10 mM CaCl_2_, and (D) various concentrations of PFOA with an addition of 100 mM CaCl_2_.

Figure 7A compares t* of KPFOS and PFOA, demonstrating that at lower concentrations, the characteristic decay time of the fast fall stage of KPFOS is smaller (e.g., a shorter time is required to reach equilibrium) than that of PFOA. Nevertheless, t* of KPFBS and PFOA tend to become equal with increasing bulk concentration. t* is independent of bulk concentration equal to and above the CMC. Moreover, at lower bulk concentrations, t*s of PFOA are less dependent on bulk concentration compared to those of KPFOS. Parts B and C of Figure 7 present the effects of adding electrolytes on the characteristic decay time of the surface tension fast fall stage. Figure 7B reveals that adding NaCl reduces the fast fall stage characteristic decay time of KPFOS and makes t* less dependent on the bulk concentration. CaCl_2_ makes t* of KPFOS more dependent on the bulk concentration. In other words, in the presence of 10 and 100 mM CaCl_2_, the t* of KPFOS becomes greater in lower concentration regimes and becomes lower in higher concentration regimes. Figure 7C shows the trend of t* change in various PFOA systems, that is, in the presence and absence of electrolytes. Regardless of the valence and concentration, t* decreases until reaching the CMC for PFOA.

**Figure 7:**
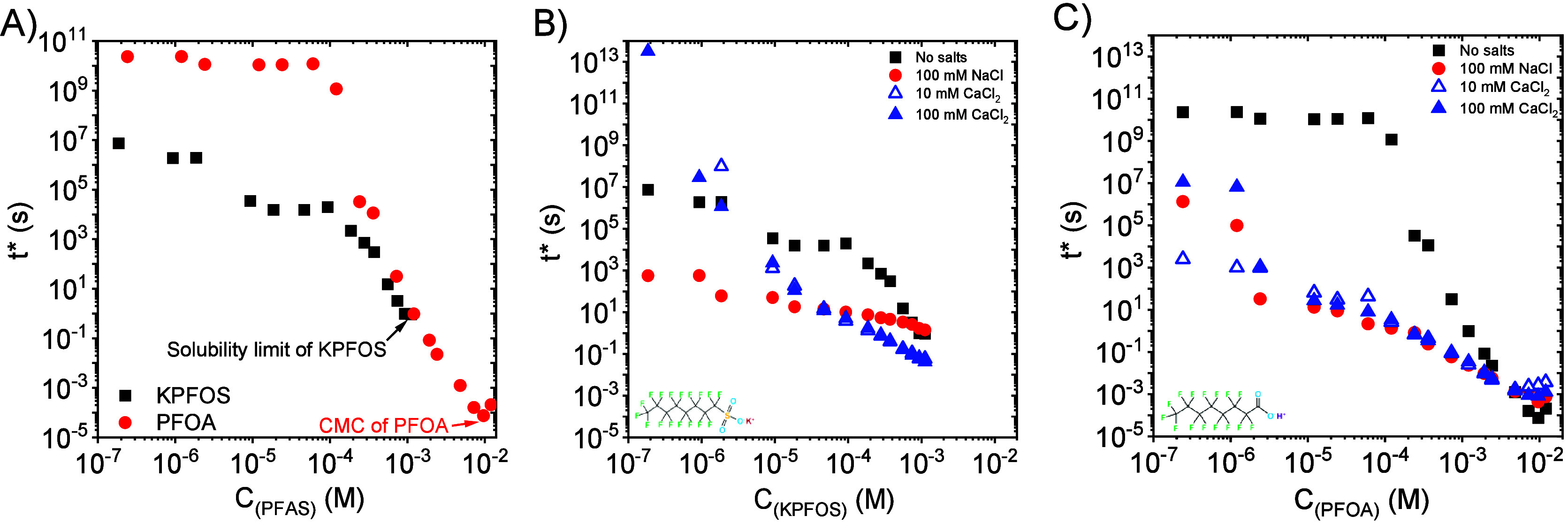
(A) Comparison of characteristic decay time of the surface tension fast fall stage of KPFOS and PFOA at various bulk concentrations in the absence of salts. (B) Effect of adding electrolytes (NaCl and CaCl_2_) on the characteristic decay time of the surface tension fast fall stage of KPFOS aqueous solutions. (C) Effect of adding electrolytes (NaCl and CaCl_2_) on the characteristic decay time of the surface tension fast fall stage of PFOA aqueous solutions.

Figure 8A shows DSTs of 0.4 mM KPFOS in the presence of various concentrations of NaCl, and Figure 8B shows the influence of various concentrations of CaCl_2_ on DSTs of 0.4 mM KPFOS; the same is shown for 0.4 mM PFOA in Figures 8C,D. Figure 8E considers the effect of various concentrations of CaCl_2_ on DSTs of 0.4 mM KPFBS. As a reminder, the solid lines presented in Figure 8 are fittings using eq 5 (fitted parameters are listed in Tables S13–S17). At a concentration of 5–10 mM, the DST is maximally reduced for the addition of NaCl to KPFOS. Above 5–10 mM, the induction stage becomes longer even though the equilibrium surface tension of KPFOS is independent of the NaCl concentration (Figure 4). No meso-equilibrium stage is reached. Figure 8B reveals that the fast fall stage is shortened and γ_0_ – *γ*_m_ increases with increasing CaCl_2_ concentration which agrees with Figure 4. Also, the meso-equilibrium stage is reached at low CaCl_2_ concentrations.

**Figure 8:**
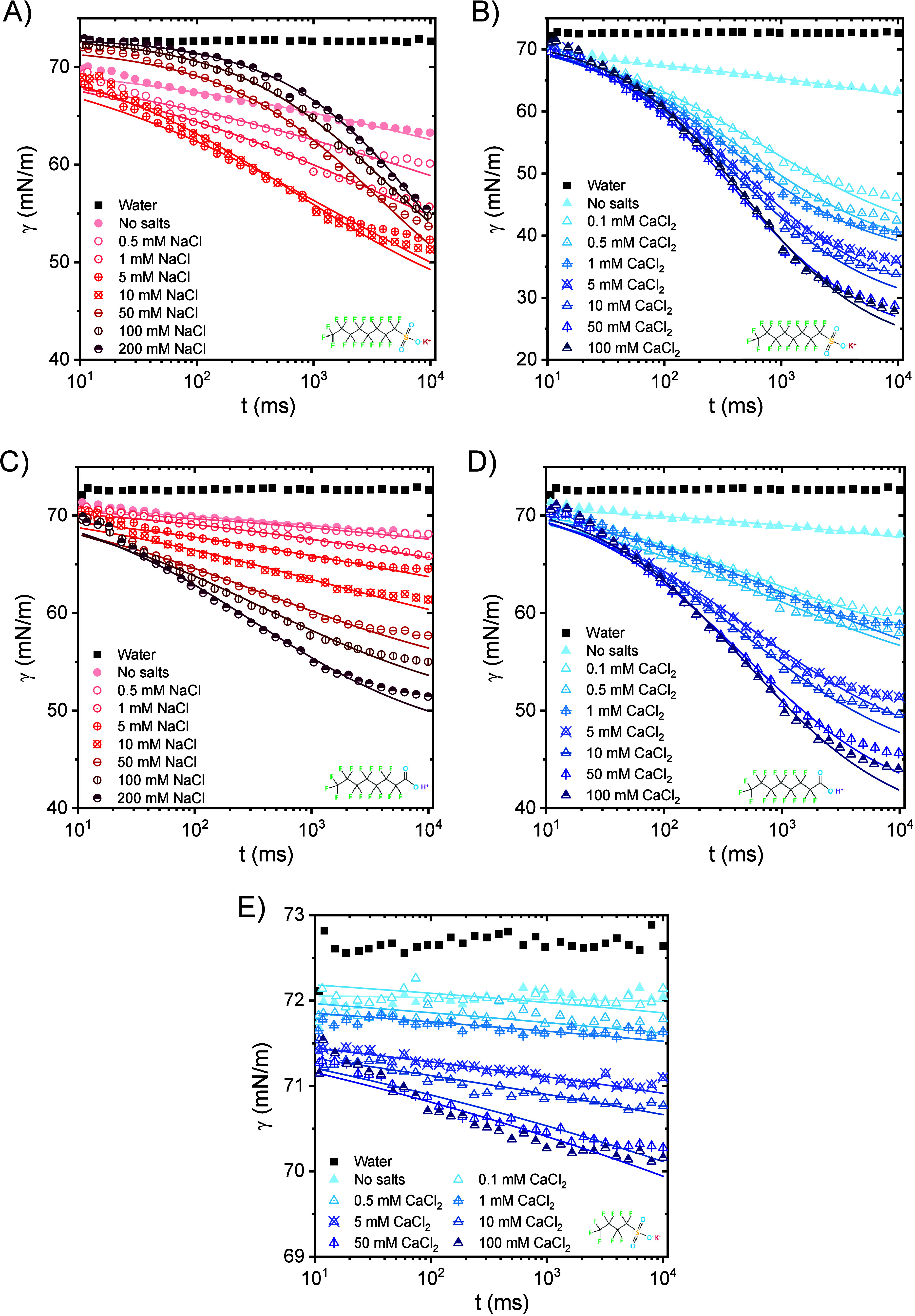
DSTs of 0.4 mM KPFOS aqueous solutions (A) with various concentrations of NaCl, and (B) with various concentrations of CaCl_2_. DSTs of 0.4 mM PFOA aqueous solutions (C) with various concentrations of NaCl, and (D) with various concentrations of CaCl_2_. (E) DSTs of 0.4 mM KPFBS aqueous solutions with various concentrations of CaCl_2_.

Differing from KPFOS, the DST of PFOA decreases monotonically with increasing NaCl concentration, and a meso-equilibrium stage appears. The decrease in DST is also monotonic with added CaCl_2_, and Figure 8D indicates that the meso-equilibrium stage also occurs at low CaCl_2_ concentrations. For KPFBS, when a small amount of CaCl_2_ (0.1–1 mM) is present, the dynamic surface tension is slightly reduced, the induction stage is difficult to observe, and no meso-equilibrium stage is found. With increasing CaCl_2_ concentration, γ_0_ – *γ*_m_ increases, the induction stage is shortened, and the meso-equilibrium stage is still absent.

Parts A and B of Figure 9 present the influence of electrolyte concentrations on the characteristic decay time (t*) of the fast fall stage for the surface tension of 0.4 mM KPFOS and 0.4 mM PFOA aqueous solutions, respectively. CaCl_2_ has no significant impact on t* for both KPFOS and PFOA; however, the NaCl concentration has a meaningful effect on t*. The minimum t* for KPFOS is observed when using 10 mM NaCl, indicating that this concentration is the most effective to reduce KPFOS dynamic surface tension in a short time scale (most effective for mass transport of molecules to the interface). t* of 0.4 mM PFOA monotonically decreases with increasing NaCl concentration. Although no significant influence of increasing CaCl_2_ concentrations on t* is found for long-chain PFAS, t* of the short-chain PFBS is decreased by increasing the concentration of CaCl_2_ as seen in Figure 9C.

**Figure 9:**
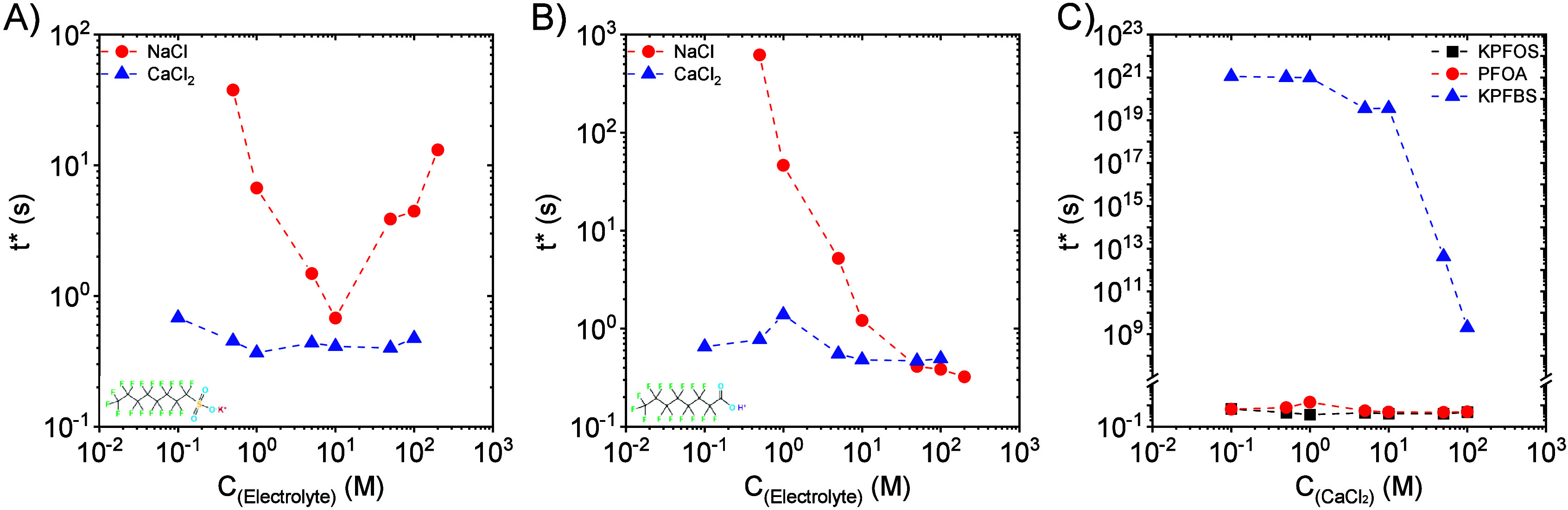
(A) Effect of electrolyte (NaCl and CaCl_2_) concentrations on the characteristic decay time of the surface tension fast fall stage for 0.4 mM KPFOS aqueous solutions. (B) Effect of electrolyte (NaCl and CaCl_2_) concentrations on the characteristic decay time of the surface tension fast fall stage for 0.4 mM PFOA aqueous solutions. (C) Comparison of the influence of various concentrations of CaCl_2_ on the characteristic decay time of the fast fall stage for surface tension of 0.4 mM PFAS aqueous solutions.

### 3.3 Diffusion

The adsorption mechanism of surfactant molecules in the absence of micelles is illustrated in Figure 10A, which introduces the concept of subsurface, which is found a few molecular diameters below the interface. The adsorption–diffusion model involves two stages: first the molecules arrive at the subsurface from the bulk driven by a concentration or surface tension gradient, followed by adsorption at the interface.^54,55,60,69^ Equation 6, the classic Ward–Tordai model, is typically used to estimate the diffusion coefficient (*D*) with the assumption that the diffusion rate of molecules from the bulk to subsurface governs the adsorption process or adsorption rate, i.e., no energy barrier present when the molecules are transported from the subsurface to the interface.^54^

**Figure 10:**
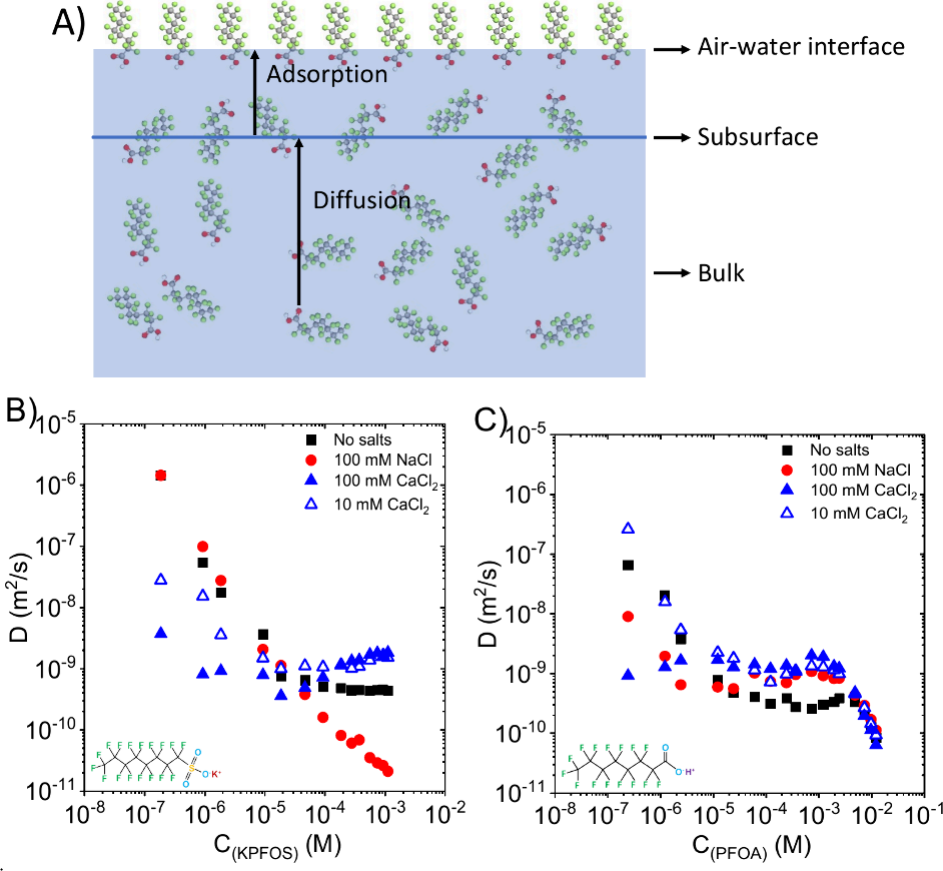
(A) Adsorption–diffusion mechanism of PFAS molecules to the air–water interface. The estimated Fickian diffusion coefficient (*D*) of (B) KPFOS and (C) PFOA as a function of their bulk concentration with and without the addition of electrolytes (NaCl and CaCl_2_).

The estimated *D* values of KPFOS and PFOA with and without adding electrolyte are presented in Figure 10B,C. Typically, the change of *D* vs PFAS bulk concentrations has three stages. The first stage occurs within the lower concentration regime. In this stage, *D* decreases when the bulk PFAS concentration increases. Mohammadi et al.^70^ reported the reduction of *D* of asphaltenes with an increase in bulk concentration and Stasiak et al.^71^ reported the same trend of *D* for commercial surfactant Nafol 810D. Yoshimura et al.^54^ used the Ward–Tordai model to study the diffusion of partially fluorinated surfactants, and their results also show that the diffusion constant is reduced by increasing the bulk concentration. This trend suggests that when the system is dilute, PFAS molecules can more easily find open positions at the subsurface,^72^ i.e., the assumption of no energy barrier from the subsurface to the surface is not correct, or a slower migration of surfactant molecules occurs from the bulk to interface due to the increase of intermolecular interactions.^70^

In the second stage, the measured diffusion coefficient becomes independent of the bulk PFAS concentration. The third stage takes place as the system approaches the CMC, where an additional energy barrier is present and likely governs adsorption from the subsurface to the interface. In this regime, the interfacial layer becomes increasingly saturated and further adsorption requires rearrangement to accommodate more molecules on the tightly packed interface. As a result, the diffusion coefficients estimated using the Ward–Tordai model begin to decrease. It is not necessarily due to reduced diffusivity in the bulk but due to kinetic limitations at the interface that are not captured by the model. In our study, the third stage is only found for PFOA as seen in Figure 10C. For KPFOS systems, the influences of monovalent and divalent salts are quite different. In the presence of 100 mM NaCl, *D* decreases monotonically in the studied KPFOS concentration range. It is not clear if the relatively low CMC in this system (∼0.4 mM) is truncating the second stage. However, upon addition of 100 mM CaCl_2_, *D* becomes less dependent on the KPFOS concentration, and two stages are still observed. *D* decreases at low KPFOS concentrations but is slightly increased in the second stage rather than hitting a plateau. In addition, at the higher CaCl_2_ concentration, *D* depends less on KPFOS bulk concentration at low concentration. For PFOA, the addition of electrolyte makes *D* less dependent on PFOA bulk concentrations until reaching the CMC (Figure 10C).

The effect of electrolyte concentration on the diffusion constant is shown in Figure 11A,B. When increasing the electrolyte concentrations to around 5–10 mM, the *D* values of both KPFOS and PFOA increase. The highest diffusion rate of KPFOS is obtained when 5 mM NaCl is presented (Figure 11A), and above 5 mM, *D* decreases due to the formation of micelles. In the case of CaCl_2_, *D* of KPFOS reaches a plateau when the CaCl_2_ concentration is higher than 10 mM. *D* of PFOA also reaches a plateau above 10 mM; however, in this case regardless of salt type (Figure 11B). Figure 11C compares the *D* values of 0.4 mM KPFOS, PFOA, and KPFBS in the presence of CaCl_2_ with various concentrations. *D* of KPFBS also depends on the CaCl_2_ concentration, and when the CaCl_2_ concentration is greater than 10 mM, *D* of KPFBS also reaches a plateau. It is evident that increasing the electrolyte concentration enhances the Fickian diffusion coefficient of PFAS below their CMC. This behavior is likely due to the neutralization or screening of the anionic headgroups by excess cations, which reduces the interaction between the PFAS and the bulk and, thereby, enhances the diffusivity.

**Figure 11:**
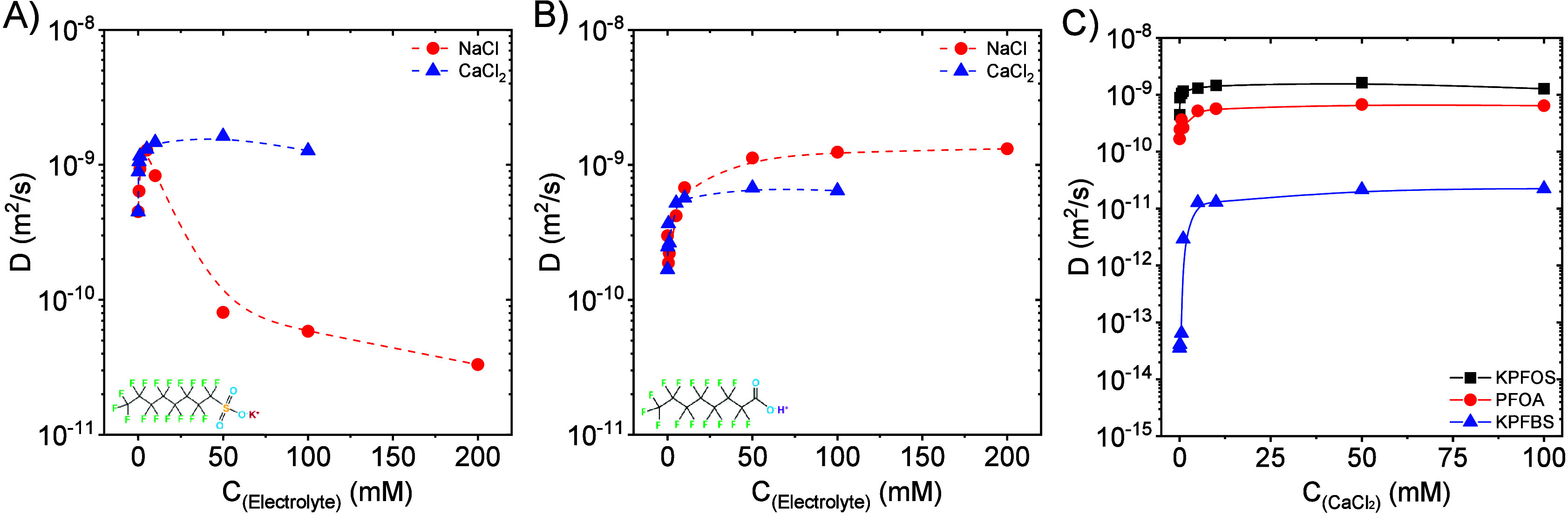
(A) Effect of electrolyte (NaCl and CaCl_2_) concentrations on the Fickian diffusion coefficient (*D*) of 0.4 mM KPFOS in aqueous solutions. (B) Effect of electrolyte (NaCl and CaCl_2_) concentrations on the Fickian diffusion coefficient (*D*) of 0.4 mM PFOA aqueous solutions. (C) Comparison of the Fickian diffusion coefficient (*D*) of 0.4 mM PFAS aqueous solutions in the presence of various concentrations of CaCl_2_.

## 4 Conclusions

In this study, surface tensions of PFAS aqueous solutions in the absence and presence of electrolytes were examined by using different tensiometries including Wilhelmy plate, pendant drop, and bubble pressure methods. Three common PFAS, KPFOS, PFOA, and KPFBS, were studied. Neither KPFOS nor KPFBS in the absence of electrolyte exhibited a CMC within the studied concentrations while PFOA had a CMC (∼9.7 mM) within the studied concentrations. At a specific concentration, 0.4 mM, the equilibrium surface tensions of PFAS follow the order of: KPFOS < PFOA < KPFBS. Equilibrium surface tensions as a function of bulk concentration were fitted with Gibbs, and Extended Langmuir isotherms, and our results indicate that the maximum surface concentrations of long-chain PFAS (KPFOS and PFOA) are very similar to that of short-chain PFAS (KPFBS) according to the Gibbs isotherm; however, short-chain PFAS have a slightly higher maximum surface concentration according to the Extended Langmuir model.

In the presence of electrolytes, the surface tension is reduced for all of the PFAS studied, consistent with previously reported findings. The CMC shifts to lower concentrations upon the addition of salt, except for short-chain PFAS (KPFBS). A smaller CMC for KPFOS is obtained when adding 100 mM CaCl_2_ compared to adding 10 mM CaCl_2_. Furthermore, the addition of electrolytes enhances the PFAS adsorption density at the air–water interface.

The short-time adsorption behavior of PFAS was investigated by estimating the diffusion coefficients from surface tension measurements obtained by using a bubble pressure tensiometer. Determination of t* and *D* for KPFOS, PFOA, and KPFBS systems using this approach is novel among currently available literature to the best of the authors knowledge. This work also addresses a notable gap in the literature by systematically examining how electrolyte valency and concentration affect the interfacial diffusivity of PFAAs, offering comparative insight into ion-specific effects on adsorption kinetics. According to the classic Ward–Tordai model, the diffusion coefficient of long-chain PFAS is dependent on its bulk concentration, where three stages of diffusion coefficients were observed. At the first stage, the diffusion coefficient decreases with an increase in the bulk concentration. When entering the second stage, the diffusion coefficient is independent of the bulk concentration. Nearing the CMC, the diffusion coefficient again decreases with an increase in the bulk concentration. At identical bulk concentrations, the diffusion coefficient of PFAS follows the order of: KPFBS < PFOA < KPFOS. In terms of the electrolyte effect, the diffusion coefficient of all PFAS tested in this work increase in the presence of CaCl_2_, and the increases rate becomes slower until approaching a plateau with increasing CaCl_2_ concentration. However, a maximum diffusion coefficient of KPFOS was observed at a specific NaCl concentration (5 mM), above which the diffusion coefficient of KPFOS decreases.

## Supplementary Material



## Notes

Equations for Gibbs free energy of micellization (Δ*G*_mic_); measurement uncertainty calculations; Gibbs isotherm fitted parameters; third-order polynomial fitted parameters; extended Langmuir isotherm fitted parameters, fitted parameters of eq 5; pendant drop profiles; additional instrumental details for the rising bubble method and bubble pressure tensiometry (PDF)
