# Supplementary material for: Influence of Electrolytes on the Air–Water Interfacial Properties of Perfluoroalkyl Acids (PFAAs)
Source: Langmuir. 2026 Feb 23;42(9):6623–36. doi: 10.1021/acs.langmuir.5c04005 (PMC12980842; doi:10.1021/acs.langmuir.5c04005)
Supplement: Supplementary file 1 [file la5c04005_si_001.pdf]

Supporting Information

for

Influence of Electrolytes on the Air-Water Interfacial Properties of Perfluoroalkyl Acids (PFAAs)

Muchu Zhou[1,2], Hayden B. McCray[1], Bor-Jier (Ben) Shiau[3], Brian P. Grady[1], Reza Foudazi[1,*]

[1] School of Sustainable Chemical, Biological and Materials Engineering, The University of Oklahoma, Norman, OK 73019, USA

[2] Chemical Sciences Division, Oak Ridge National Laboratory, Oak Ridge, TN 37831, USA

[3] Mewbourne School of Petroleum and Geological Engineering, The University of Oklahoma, Norman, OK 73019, USA

[*] Corresponding Author. Email: rfoudazi@ou.edu

Equations for Gibbs free energy of micellization (ΔG_{mic})

The Gibbs free energy of micellization (ΔG_{mic}) is the net free-energy gain for transferring a free surfactant molecule and its counterion from the bulk aqueous solution to a micelle. *Equation (S1)* below shows the relationship between the CMC of ionic surfactants and ΔG_{mic}.

$$\Delta G_{mic} = (2 - \alpha)RTln(X_{CMC}) \qquad \textit{Equation (S1)}$$

where α is the degree of counterion dissociation, and X_{CMC} is the mole fraction of surfactants at the CMC. The Corrin–Harkins equation, $ln(X_{CMC}) = A - \beta ln(X_C)$, can be used to obtain the α. X_C is the total concentration of free counterion (mole fraction) in the solution at the CMC, and $\beta = 1 - \alpha$ is the degree of counterion binding. Without adding electrolytes, $X_{CMC} = X_C$ and $\Delta G_{mic}/RT = A = (1 + \beta)ln(X_{CMC})$.

The *Equation (S2)* considers different contributions to the total ΔG_{mic}.

$$\Delta G_{mic} = \Delta G_{tr} + \Delta G_{int} + \Delta G_{pack} + \Delta G_{st} + \Delta G_{elec} + \Delta G_{ent} - \beta kTln(X_c e) - kT \qquad \textit{Equation (S2)}$$

where ΔG_{tr} is the transfer free energy, ΔG_{int} is the interfacial free energy, ΔG_{pack} is the packing free energy, ΔG_{st} is the steric free energy, ΔG_{elec} is the electrostatic free energy, ΔG_{ent} is the entropic free energy, and k is the Boltzmann constant. ΔG_{pack} is associated with the formation of hydrophobic core in the micelles, whereas ΔG_{ent} is linked to the hydrophilic shell of the micelles. In *Equation (S2)*, $-\beta kTln(X_c e)$ is the translational entropy lost by the counterions upon binding onto the charged surface of micelles.

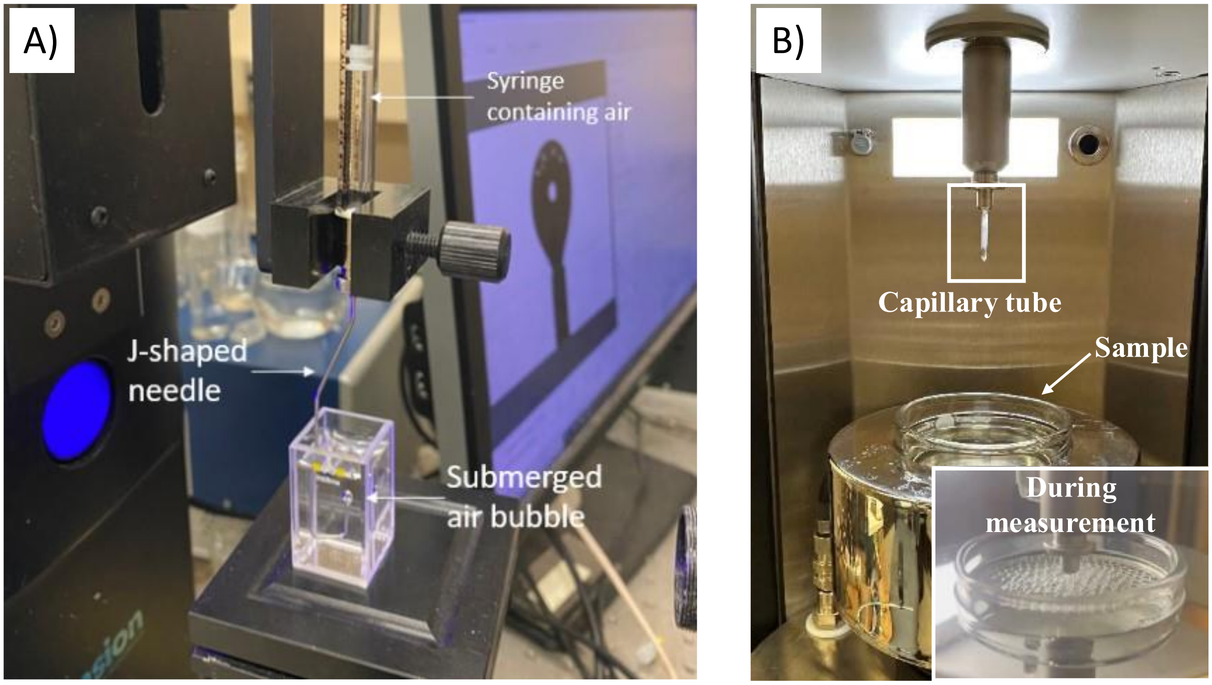

Figure S1. **A)** The rising bubble method by using the J-shaped needle. **B)** Bubble pressure tensiometer used in this study, the capillary tube and sample holder are highlighted. The bubbles are generated during the surface tension measurement.

Measurement uncertainty calculations

The combined standard uncertainty was calculated using the law of propagation of uncertainty, as described by the following equation, to evaluate the impact of measurement uncertainty on the reported results:

$$u_\sigma(y) = \sqrt{\sigma_y^2 + [\frac{\partial y}{\partial x_1}u(x_1)]^2 + [\frac{\partial y}{\partial x_2}u(x_2)]^2 + \cdots + [\frac{\partial y}{\partial x_n}u(x_n)]^2} \qquad \textit{Equation (S3)}$$

where $u_\sigma(y)$ represents the combined standard uncertainty of the measured dependent variable y (i.e., surface tension), σ_y is the standard deviation of y based on repeated measurements, and $u(x_i)$ denotes the uncertainty associated with the instrument or apparatus used to measure the independent variable x_i. The uncertainties for each instrument are summarized in **Table S1**.

Table S1. $u(x)$ of the equipment/apparatus.

**Equipment**	$\boldsymbol{u(x_i)}$
Balance	±0.0001 g
Graduated cylinder	±0.1 mL
Wilhelmy plate tensiometer (Data physics, DCAT 25)	±0.001 mN/m
Wilhelmy plate tensiometer (Data physics, DCAT 25)	±20 µg
Pendant drop tensiometer (Attension KSV Instruments, Biolin Scientific, Finland)	±0.01 mN/m
Pendant drop tensiometer (Attension KSV Instruments, Biolin Scientific, Finland)	±0.01 µL
Bubble pressure tensiometer (KRÜSS, BP100)	±0.1 Pa
Bubble pressure tensiometer (KRÜSS, BP100)	±0.005 mm
Bubble pressure tensiometer (KRÜSS, BP100)	±0.10-0.15 mN/m

As an example, we consider surface tension measurements by bubble pressure tensiometer (KRÜSS, BP100). The basic formula for calculating surface tension using the bubble pressure tensiometer is shown below:

$$\gamma = \frac{\Delta P_{max} \cdot r}{2} \qquad \textit{Equation (S4)}$$

where γ is the surface tension (mN/m), ΔP_{max} is the maximum pressure difference (Pa), r is the radius of the capillary tip (m). From *Equation (S4)*, we can obtain the partial derivatives:

$$\frac{\partial \gamma}{\partial \Delta P_{max}} = \frac{r}{2}$$ *Equation (S5)*

$$\frac{\partial \gamma}{\partial r} = \frac{\Delta P_{max}}{2}$$ *Equation (S6)*

Then, substituting these into the *Equation (S3)* gives:

$$u_\sigma(\gamma) = \sqrt{(\frac{r}{2} \cdot u(\Delta P_{max}))^2 + (\frac{\Delta P_{max}}{2} \cdot u(r))^2 + \sigma_\gamma^2}$$ *Equation (S7)*

Since the capillary radius r (0.228 mm) is precisely known, $u(r)$ is negligible. Then, *Equation (S7)* simplifies to:

$$u_\sigma(\gamma) = \sqrt{(\frac{r}{2} \cdot u(\Delta P_{max}))^2 + \sigma_\gamma^2}$$ *Equation (S8)*

As an example, consider 0.4 mM KPFOS aqueous solutions. The dynamic surface tension at 9926.15 ms is 63.28±0.22 mN/m based on the measurements; therefore, $\sigma_y = 0.22$ mN/m. The pressure uncertainty, $u(\Delta P_{max})$, is ±0.1 Pa; therefore, *Equation (S8)* becomes:

$$u_\sigma(\gamma) = \sqrt{(\frac{0.000228}{2} \cdot 0.1)^2 + 0.22^2} = 0.22 \text{ mN/m}$$ *Equation (S9)*

Therefore, our calculation indicates that the combined standard uncertainty $u_\sigma(\gamma)$ for using bubble pressure tensiometer (KRÜSS, BP100) for 0.4 mM KPFOS aqueous solutions is around 0.22 mN/m, which is similar to the standard deviation $\sigma_y = 0.22$ mN/m (error bar) based on the measurement using bubble pressure tensiometer (KRÜSS, BP100).

For measuring the surface tension by pendant drop tensiometer (Attension KSV Instruments, Biolin Scientific, Finland), the software fits the droplet profile using the Young–Laplace equation, which relates the curvature of the droplet to the surface tension:

$$\gamma = f(\rho, g, R, \Delta h)$$ *Equation (S10)*

where ρ is the density of the liquid, g is the gravitational constant, R is the drop radius of curvature, and Δh is the height difference in shape profile. The surface tension is automatically calculated from the image using known physical parameters. Uncertainty in the pendant drop method arises from instrument resolution and calibration, including camera pixel size and optical distortion, fit errors in droplet shape matching, density measurement error (usually negligible if taken from known values), and reproducibility between drops or over time. Since the shape-fitting algorithm is complex and partial derivatives are difficult to extract, we use the law of propagation of uncertainty in a practical, empirical form:

$$u_\sigma(\gamma) = \sqrt{(u(instrument))^2 + \sigma_\gamma^2}$$ *Equation (S11)*

Considering 0.4 mM PFOA aqueous solution as an example, the equilibrium surface tension is 62.542±0.095 mN/m from pendant drop tensiometry (Attension KSV Instruments, Biolin Scientific, Finland); thus, $\sigma_y = 0.095$ mN/m in *Equation (S11)*:

$$u_\sigma(\gamma) = \sqrt{(0.01)^2 + (0.095)^2} = 0.096 \text{ mN/m} \qquad \textit{Equation (S12)}$$

Therefore, our calculation indicates that the combined standard uncertainty $u_\sigma(\gamma)$ for using pendant drop tensiometer (Attension KSV Instruments, Biolin Scientific, Finland) for 0.4 mM PFOA aqueous solutions is around 0.096 mN/m, which is in the same order of magnitude as the standard deviation $\sigma_y = 0.095$ mN/m (error bar).

Since the rising bubble method uses the same instrument and software to measure the surface tension, we can conclude that the combined standard uncertainty for using rising bubble method is similar to the standard deviation due to the repeated measurements.

In our previous work,[1] we have shown that the combined standard uncertainty for using Wilhelmy plate tensiometer (Dataphysics, DCAT 25) has no significant difference compared to the standard deviation. Therefore, we can use **Table 2** to summarize the systematic differences of surface tension obtained from those used methods.

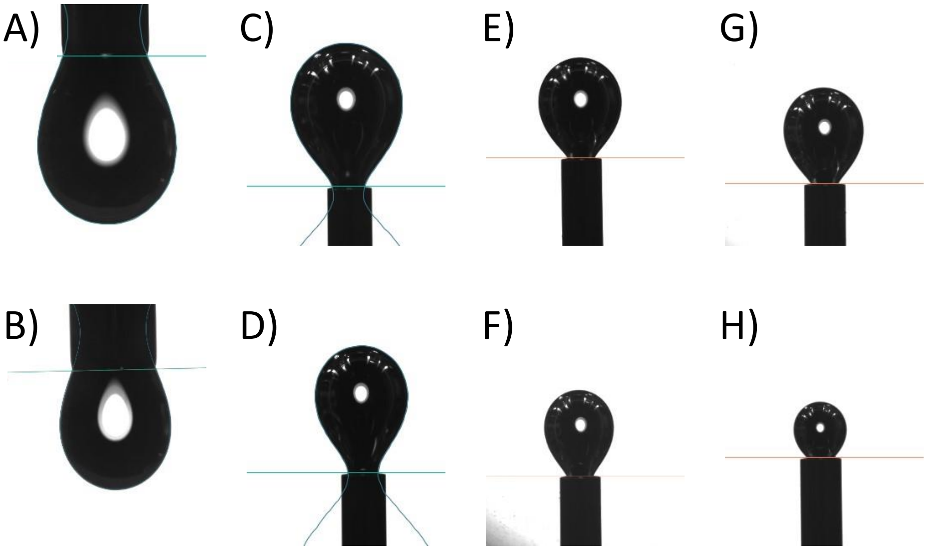

Figure S2. Pendant drops of **A)** 0.4 mM, and **B)** 2.4 mM PFOA aqueous solutions in air. Rising air bubbles in **C)** 0.4 mM, and **D)** 2.4 mM PFOA aqueous solutions. Rising air bubbles in **E)** 0.4 mM, and **F)** 2.4 mM PFOA aqueous solutions containing 10 mM $CaCl_2$. And rising air bubbles in **G)** 0.4 mM, and **H)** 2.4 mM PFOA aqueous solutions containing 100 mM $CaCl_2$.

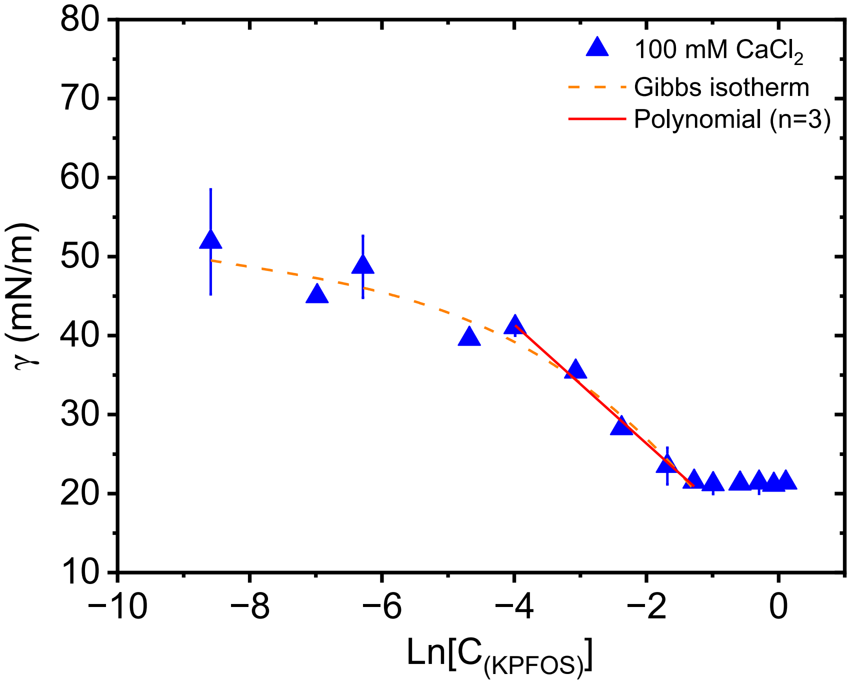

Figure S3. Gibbs isotherm fit and polynomial fit with the third order to the surface tension of KPFOS in the presence of 100 mM $CaCl_2$. The concentration of KPFOS is in mM.

Table S2. Gibbs isotherm fitted parameters for PFAAs solutions with and without salts.

	Slope (mN/m)	R^2
KPFOS[1]	-9.736±0.929	0.95644
KPFOS+100 mM NaCl	-5.295±0.008	0.99980
KPFOS+10 mM $CaCl_2$	-6.560±0.174	0.99507
KPFOS+100 mM $CaCl_2$	-7.531±0.422	0.99067
PFOA (Pendant drop)	-16.38021±0.8164	0.98773
PFOA (Rising bubble)	-13.86149±0.7381	0.98327
PFOA	-9.5889±0.41198	0.98545
PFOA+100 mM NaCl	-13.36559±0.59478	0.99214
PFOA+10 mM $CaCl_2$	-9.16678±0.4597	0.98514
PFOA+100 mM $CaCl_2$	-8.87088±0.17826	0.99758
KPFBS[1]	-9.535±1.807	0.93299
KPFBS+100 mM $CaCl_2$	-6.5464±0.25004	0.98846

Table S3. Third-order polynomial fitted parameters for PFAAs solutions with and without salts.

	a	b	c	d	R^2
KPFOS[1]	-0.03909	-1.03376	-10.26362	35.93399	0.99146
KPFOS+100 mM NaCl	-0.01511	-0.25487	-4.67843	39.26547	0.99266
KPFOS+10 mM $CaCl_2$	-0.01028	-0.40411	-8.24873	19.60713	0.9987
KPFOS+100 mM $CaCl_2$	-0.06168	-1.4889	-13.33373	5.74739	0.97056
PFOA (Pendant drop)	0.24684	-1.97506	-14.87652	49.9029	0.99735
PFOA (Rising bubble)	0.89109	-0.82395	-15.55976	47.55947	0.99239
PFOA	0.02043	-0.82296	-10.22422	-43.99098	0.99653
PFOA+100 mM NaCl	-0.04853	-1.14351	-9.96539	40.79983	0.99629
PFOA+10 mM $CaCl_2$	-0.00701	-0.67578	-9.5414	35.49379	0.99825
PFOA+100 mM $CaCl_2$	0.00919	-0.41709	-9.40368	28.49137	0.9979
KPFBS[1]	-0.10027	-0.64343	-1.50557	69.8844	0.98189
KPFBS+100 mM $CaCl_2$	-0.00168	-0.41138	-3.59506	63.74608	0.98754

Table S4. Extended Langmuir isotherm fitted parameters for PFAAs solutions with and without salts.

	a ($\Gamma_m \times 10^{10}\ mol/cm^2$)	b (K_L, $(L/mmol)^n$)	c $(1-n)$	R^2
KPFOS[1]	3.28894±0.19123	1.57887±0.22085	0.57756±0.01709	0.99904
KPFOS+100 mM NaCl	5±16.57856	0.55922±2.87746	0.9257±0.10618	0.86358
KPFOS+10 mM $CaCl_2$	13±4.83008	0.34118±0.16893	0.87748±0.01063	0.9987
KPFOS+100 mM $CaCl_2$	15.33402±2.7923	0.54524±0.15	0.65273±0.01829	0.99873
PFOA (Pendant drop)	7.62689±0.59391	0.53846±0.07407	0.3493±0.04281	0.99708
PFOA (Rising bubble)	5.56093±0.39111	0.70862±0.09819	0.451±0.03386	0.99718
PFOA	3.11794±0.14781	2.122±0.40988	0.3967±0.0568	0.98921
PFOA+100 mM NaCl	12.00155±0.49952	0.50368±0.03417	0.63909±0.00793	0.99959
PFOA+10 mM $CaCl_2$	6.44469±0.32807	1.56633±0.23423	0.62599±0.02339	0.99523
PFOA+100 mM $CaCl_2$	6.08783±0.01165	6.18357±0.08648	0.79353±0.00145	0.99994
KPFBS[1]	3.89226±0.20864	0.08868±0.00393	0.29193±0.02836	0.99807
KPFBS+100 mM $CaCl_2$	3.34889±0.15737	0.71364±0.07156	0.5272±0.02989	0.99236

Table S5. Fitted parameters of *Equation (5)* for KPFOS with various bulk concentrations.

PFAS	Concentration (mM)	Salt	Concentration (mM)	t* [s]	n
KPFOS	1.2	-	-	0.936985	0.295203
	1			0.976069	0.298348
	0.8			3.284151	0.269974
	0.6			15.32155	0.242519
	0.4			307.6646	0.20512
	0.3			711.7598	0.199193
	0.2			2203.413	0.203915
	0.1			19801.31	0.230556
	0.05			15522.89	0.309352
	0.02			15550.16	0.352767
	0.01			35288.96	0.355652
	0.002			1951989	0.281189
	0.0009			1893031	0.139115
	0.0002			7326244	0.099944

Table S6. Fitted parameters of *Equation (5)* for KPFOS with various bulk concentrations in the presence of 100 mM NaCl as the background ions.

PFAS	Concentration (mM)	Salt	Concentration (mM)	t* [s]	n
KPFOS	1.2	NaCl	100	1.407726	0.667961
	1			1.663604	0.677291
	0.8			2.616811	0.656011
	0.6			3.388555	0.674606
	0.4			4.605189	0.693226
	0.3			5.462521	0.733708
	0.2			7.479746	0.754986
	0.1			10.12929	0.829493
	0.05			14.19215	0.856717
	0.02			18.20482	0.937932
	0.01			50.89052	0.973583
	0.002			60.93649	1.653996
	0.0009			575.1623	0.82458
	0.0002			562.794	0.742586

Table S7. Fitted parameters of *Equation (5)* for KPFOS with various bulk concentrations in the presence of 10 mM $CaCl_2$ as the background ions.

PFAS	Concentration (mM)	Salt	Concentration (mM)	t* [s]	n
KPFOS	1.2	$CaCl_2$	10	0.058154	0.599024
	1			0.060732	0.631623
	0.8			0.108082	0.624051
	0.6			0.174151	0.682401
	0.4			0.399488	0.725241
	0.3			0.744077	0.75382
	0.2			1.340712	0.844794
	0.1			3.862268	0.784629
	0.05			14.51199	0.742859
	0.02			184.8321	0.535795
	0.01			1300.935	0.425964
	0.002			98814094	0.188716
	0.0009			2.03E+14	0.095004
	0.0002			2.04E+14	0.114088

Table S8. Fitted parameters of *Equation (5)* for KPFOS with various bulk concentrations in the presence of 100 mM $CaCl_2$ as the background ions.

PFAS	Concentration (mM)	Salt	Concentration (mM)	t* [s]	n
KPFOS	1.2	$CaCl_2$	100	0.042337	0.765254
	1			0.066398	0.778939
	0.8			0.088044	0.790226
	0.6			0.165615	0.772025
	0.4			0.431408	0.784541
	0.3			0.831928	0.78875
	0.2			1.716451	0.846052
	0.1			5.162397	0.876909
	0.05			11.93616	1.039464
	0.02			113.6201	0.714398
	0.01			2324.632	0.486908
	0.002			1182339	0.339386
	0.0009			28938763	0.282
	0.0002			3.21E+13	0.142158

Table S9. Fitted parameters of *Equation (5)* for PFOA with various bulk concentrations.

PFAS	Concentration (mM)	Salt	Concentration (mM)	t* [s]	n
PFOA	12.0	-	-	0.000208	0.374562
	9.7			7.59E-05	0.29338
	7.3			0.000161	0.319094
	4.8			0.001253	0.316266
	2.4			0.022664	0.236619
	1.9			0.084217	0.250941
	1.2			0.970405	0.218217
	0.8			31.75601	0.197091
	0.4			11353.17	0.181282
	0.24			32606.63	0.192821
	0.12			1.16E+09	0.203832
	0.06			1.2E+10	0.138533
	0.024			1.1E+10	0.069483
	0.012			1.09E+10	0.042743
	0.002			1.13E+10	0.095545
	0.001			2.36E+10	0.094187
	0.0002			2.33E+10	0.049778

Table S10. Fitted parameters of *Equation (5)* for PFOA with various bulk concentrations in the presence of 100 mM NaCl as the background ions.

PFAS	Concentration (mM)	Salt	Concentration (mM)	t* [s]	n
PFOA	12.0	NaCl	100	0.000739	0.844073
	9.7			0.000434	0.641668
	7.3			0.00162	0.999556
	4.8			0.00133	0.573708
	2.4			0.005766	0.514987
	1.9			0.009557	0.464306
	1.2			0.023531	0.480504
	0.8			0.058918	0.481423
	0.4			0.240809	0.463291
	0.24			0.822758	0.441668
	0.12			1.342068	0.51217
	0.06			2.144273	0.581777
	0.024			8.829927	0.6663
	0.012			13.30602	0.753043
	0.002			32.57653	1.240202
	0.001			98263.12	0.17911
	0.0002			1331681	0.179315

Table S11. Fitted parameters of *Equation (5)* for PFOA with various bulk concentrations in the presence of 10 mM $CaCl_2$ as the background ions.

PFAS	Concentration (mM)	Salt	Concentration (mM)	t* [s]	n
PFOA	12.0	$CaCl_2$	10	0.001287	0.644817
	9.7			0.000812	0.54829
	7.3			0.000903	0.643506
	4.8			0.00145	0.881065
	2.4			0.005641	0.484002
	1.9			0.010913	0.512387
	1.2			0.034578	0.520676
	0.8			0.097325	0.544836
	0.4			0.34461	0.582458
	0.24			0.675052	0.650134
	0.12			3.433214	0.688356
	0.06			8.010113	0.585872
	0.024			16.86459	0.608654
	0.012			27.53452	0.579298
	0.002			1103.269	0.203573
	0.001			6558841	0.105982
	0.0002			11219484	0.059169

Table S12. Fitted parameters of *Equation (5)* for PFOA with various bulk concentrations in the presence of 100 mM $CaCl_2$ as the background ions.

PFAS	Concentration (mM)	Salt	Concentration (mM)	t* [s]	n
PFOA	12.0	$CaCl_2$	100	0.003693	0.539617
	9.7			0.002667	0.57521
	7.3			0.002314	0.522116
	4.8			0.001648	0.510832
	2.4			0.004829	0.504959
	1.9			0.009543	0.487354
	1.2			0.025154	0.54712
	0.8			0.088324	0.581748
	0.4			0.438901	0.658752
	0.24			0.679054	0.692247
	0.12			2.614235	0.727653
	0.06			42.39068	0.617623
	0.024			30.17337	0.738094
	0.012			64.50514	0.663632
	0.002			983.6452	0.415032
	0.001			997.9108	0.427407
	0.0002			2504.572	0.183129

Table S13. Fitted parameters of *Equation (5)* for 0.4 mM KPFOS aqueous solutions with various concentrations of NaCl as the background ions.

PFAS	Concentration (mM)	Salt	Concentration (mM)	t* [s]	n
KPFOS	0.4	NaCl	0	307.6646245	0.205120493
			0.5	37.70146658	0.222606044
			1	6.69460108	0.282595212
			5	1.484229733	0.337885631
			10	0.678251157	0.445889963
			50	3.87788525	0.623272862
			100	4.453508597	0.642921361
			200	13.11767727	0.656172306

Table S14. Fitted parameters of *Equation (5)* for 0.4 mM KPFOS aqueous solutions with various concentrations of $CaCl_2$ as the background ions.

PFAS	Concentration (mM)	Salt	Concentration (mM)	t* [s]	n
KPFOS	0.4	$CaCl_2$	0	307.6646245	0.205120493
			0.1	0.681613655	0.557320632
			0.5	0.452532006	0.643433695
			1	0.367505338	0.692631112
			5	0.437864718	0.696037803
			10	0.412052986	0.70767168
			50	0.399773552	0.761867957
			100	0.475131616	0.784391572

Table S15. Fitted parameters of *Equation (5)* for 0.4 mM PFOA aqueous solutions with various concentrations of NaCl as the background ions.

PFAS	Concentration (mM)	Salt	Concentration (mM)	t* [s]	n
PFOA	0.4	NaCl	0	11353.17128	0.181282428
			0.5	620.1246551	0.182537021
			1	46.41882951	0.235518285
			5	5.213302676	0.24461233
			10	1.213635718	0.310853989
			50	0.410993057	0.382948025
			100	0.384804362	0.430943271
			200	0.322027678	0.515041321

Table S16. Fitted parameters of *Equation (5)* for 0.4 mM PFOA aqueous solutions with various concentrations of $CaCl_2$ as the background ions.

PFAS	Concentration (mM)	Salt	Concentration (mM)	t* [s]	n
PFOA	0.4	$CaCl_2$	0	11353.17128	0.181282428
			0.1	0.652660471	0.453589518
			0.5	0.77614112	0.412976288
			1	1.388582144	0.409573827
			5	0.552622226	0.543718592
			10	0.481132134	0.571806486
			50	0.468817411	0.627357594
			100	0.495326027	0.67278935

Table S17. Fitted parameters of *Equation (5)* for 0.4 mM KPFBS aqueous solutions with various concentrations of $CaCl_2$ as the background ions.

PFAS	Concentration (mM)	Salt	Concentration (mM)	t* [s]	n
KPFBS	0.4	$CaCl_2$	0	4.14934E+21	0.01440141
			0.1	1.12171E+21	0.052388246
			0.5	9.95184E+20	0.046024103
			1	9.89137E+20	0.041103611
			5	3.55041E+19	0.047152941
			10	3.56231E+19	0.051907516
			50	4.2581E+12	0.075935982
			100	2044343017	0.084310727

Reference:

1. Zhou M, Chaleshtari ZA, Shiau BJ, Grady BP, Foudazi R. Air-water Interfacial Properties of Perfluorosulfonaic Acid Salts with Different Chain Lengths. *Colloids Surfaces A Physicochem Eng Asp*. 2024;694:134129. doi:10.1016/J.COLSURFA.2024.134129
